# PKCϵ-mediated phosphorylation of TRPC3 channel at S712 is essential for its inactivation during inflammatory signaling

**DOI:** 10.3389/fimmu.2025.1737430

**Published:** 2026-01-14

**Authors:** Javier Casas, Clara Meana, Gonzalo San-José, Jesús Balsinde, María A. Balboa

**Affiliations:** 1 Lipid Metabolism and Inflammation Group, IBGM, CSIC-UVA, Valladolid, Spain; 2 Departamento de Bioquímica y Biología Molecular y Fisiología, Facultad de Medicina, Universidad de Valladolid, Valladolid, Spain; 3 Departamento de Pediatría e Inmunología, Facultad de Medicina, Universidad de Valladolid, Valladolid, Spain; 4 Centro de Investigación Biomédica en Red de Diabetes y Enfermedades Metabólicas Asociadas (CIBERDEM), Madrid, Spain; 5 Bioactive Lipids and Lipidomics Core, IBGM, CSIC-UVA, Valladolid, Spain

**Keywords:** calcium, inflammation, macrophage, protein kinase C (PKC), TRPC3 channels

## Abstract

The transient receptor potential canonical 3 (TRPC3) channel plays a pivotal role in macrophage-mediated inflammatory signaling by regulating intracellular calcium dynamics. This study identifies phosphorylation at serine 712 (S712) by protein kinase C ϵ (PKCϵ) as a critical mechanism for TRPC3 inactivation. Using HEK-TLR4 cells and THP-1 human macrophages, we demonstrate that the S712A-TRPC3 mutant, which cannot be phosphorylated, exhibits altered subcellular localization, promoting persistent calcium influx, and enhanced expression of proinflammatory cytokines such as TNFα and inflammatory mediator enzyme COX2 during LPS cellular activation. Live-cell imaging and FRET assays reveal that PKCϵ, but not other PKC isoforms, translocates to endomembranes upon LPS stimulation and interacts directly with TRPC3. Pharmacological inhibition and gene silencing of PKCϵ mimic the effects of the S712A mutation, confirming its role in terminating TRPC3-mediated calcium signaling. These findings establish PKCϵ-mediated phosphorylation of TRPC3 at S712 as a key regulatory mechanism for resolving inflammatory calcium signaling in macrophages.

## 1 Introduction

Inflammation is a fundamental biological response to infection, injury, or stress, orchestrated by a complex network of signaling pathways that regulate immune cell activation, cytokine production, and tissue remodeling. While acute inflammation is essential for host defense and tissue repair, its dysregulation can lead to chronic inflammatory diseases, including metabolic disorders, autoimmune conditions, and cardiovascular pathologies. Central to the initiation and resolution of inflammatory responses is the precise control of intracellular Ca²^+^ signaling, which modulates key processes such as gene transcription, vesicle trafficking, and phagocytosis in immune cells.

Transient receptor potential (TRP) channels comprise several subfamilies, including canonical (TRPC), vanilloid (TRPV), and melastatin (TRPM) channels, which collectively regulate Ca²^+^ signaling in immune and non-immune cells (1, 2). While TRPV and TRPM channels have been implicated in macrophage activation and inflammatory responses, TRPC3 has emerged as a critical regulator of Ca²^+^ dynamics in macrophages (3), where it contributes to Toll-like receptor 4 (TLR4)-mediated signaling and the production of proinflammatory cytokines. TRPC3 can be activated by diacylglycerol (DAG), a lipid second messenger generated downstream of phospholipase C activity known to localize to the plasma membrane (4) and in intracellular compartments, including the endoplasmic reticulum (ER) by lipin-1, member of a family of phosphatidic acid (PA) phosphatase enzymes with key roles in metabolism and signaling in macrophages (5–11). We have previously described that TRPC3 activation facilitates Ca²^+^ release from internal stores and sustains cytosolic Ca²^+^ elevations necessary for inflammatory gene expression (8).

To prevent excessive or prolonged Ca²^+^ signaling, TRPC3 activity must be tightly regulated. One proposed mechanism involves phosphorylation by protein kinase C (PKC), a family of serine/threonine kinases activated by DAG and Ca²^+^. PKC isoforms are categorized into three major classes based on their structural domains and cofactor requirements: conventional (cPKC), novel (nPKC), and atypical (aPKC). Conventional isoforms (e.g., PKCα, PKCβ, PKCγ) require both Ca^2+^ and DAG for activation, reflecting their dependence on phospholipid signaling and Ca^2+^ influx. Novel isoforms (e.g., PKCδ, PKCϵ, PKCη, PKCθ) are Ca^2+^-independent but still require DAG, indicating a partial overlap in activation mechanisms with cPKCs. Atypical isoforms (e.g., PKCζ, PKCι/λ) are independent of both Ca^2+^ and DAG, relying instead on other regulatory inputs such as protein-protein interactions and phosphorylation events (12). These distinct activation profiles suggest differential roles for PKC isoforms in cellular signaling pathways. Trebak et al. (13) suggested that phosphorylation of TRPC3 by PKC is a mechanism of receptor-mediated negative regulation, by identifying Ser 712 in TRPC3 as an essential residue for PKC mediated negative regulation. However, the specific PKC isoform responsible for this modification and the functional consequences of S712 phosphorylation in the context of innate immune signaling remain poorly understood. Which PKC isoform phosphorylates TRPC3 at S712 during inflammatory signaling remains unknown. In this study, we investigate the molecular mechanisms underlying TRPC3 regulation by PKC in macrophages. We identify PKCϵ as the key isoform mediating S712 phosphorylation and demonstrate its role in modulating TRPC3 localization, Ca²^+^ signaling, and cytokine production during inflammatory responses. Our findings provide new insights into the spatial and temporal control of TRPC3 in this context.

## 2 Materials & methods

### 2.1 Cells

The human promonocytic cell line THP-1 was maintained in RPMI 1640 medium supplemented with 10 mM HEPES, 10% FBS, 100 U/ml penicillin, 100 μg/ml streptomycin, 2 mM glutamine, 1% sodium pyruvate, 1% non-essential amino acids solution and 50 μM β-mercaptoethanol at 37°C in a 5% CO_2_ humidified incubator. For differentiation to a macrophage phenotype, the cells were treated with 25 ng/ml PMA for 24 h, after which they were left to rest for 48 h (8).

HEK293 cells expressing human TLR4/MD2/CD14 (HEK-TLR4, Invivogen, Catalog #293-htlr4md2cd14) were cultured in DMEM supplemented with 2 mM glutamine, 10% fetal bovine serum (FBS), 1% sodium pyruvate, 1% non-essential aminoacid solution, 100 U/ml penicillin, 100 μg/ml streptomycin, 5 μg/ml blasticidin and 25 μg/ml hygromycin B at 37°C in a 5% CO_2_ humidified incubator. The cells were passaged twice a week by detachment with 1 mM EDTA in PBS.

### 2.2 Plasmids and mutagenesis

Human TRPC3 (eYFP-hTRPC3) and the N-terminal fragment of hTRPC3 (hTRPC3 (1– 321), eYFP-N-ter-TRPC3) expression plasmids were kindly provided by Dr. K. Groschner (University of Graz, Austria). Wild-type hTRPC3 was mutagenized to modify S712 phosphorylation by replacing Ser 712 with Ala (S712A) using the Quick-Change XL site-directed mutagenesis kit (Stratagene, La Jolla, CA, USA) with the following oligonucleotides: forward primer 5´-ATTACCTCCACCTTTCAGTCTAGTTCTgcTCCAAAATCATTTGTTTATTT-3´ and reverse primer 5´-AAATAAACAAATGATTTTGGAgcAGGAACTAGACTGAAAGGTGGAGGTAAT-3´. Mutagenesis was confirmed by sequencing. eYFP-TRPC3 variants were amplified by PCR adding 5´Xba-I and Sal-I 3´specific restriction sites and which removes copGFP gene in the plasmid and inserted into lentiviral vector pCDH-CMV-MCS-EF1α-copGFP. pBudCE4.1-vYC4er (vYC4er cameleon) was provided by Dr. M.T. Alonso from our institute (originally from Dr. W. Graier, University of Graz, Austria). Constructs expressing mCherry-PKCs were provided by Dr. Alexandra Newton, UCSD, USA. Positive control FRET plasmid pmCherry-eYFP was kindly provided by Dr. Johannes Schmid (University of Vienna, Austria) (14).

### 2.3 Gene silencing and plasmid transfection

Gene silencing was performed using INTERFERin transfection reagent (Polyplus-transfection, France) according to the manufacturer’s protocol. Briefly, THP-1 cells were seeded at a density of 2 × 10^5^ cells/mL in antibiotic-free RPMI-1640 medium supplemented with 10% FBS and allowed to equilibrate for 24 hours. 50 nM small interfering RNA (siRNA) targeting PRKCE was diluted in OPTIMEM and mixed with INTERFERin reagent at a ratio of 3 µL per 10 nM siRNA. The mixture was incubated for 10 minutes at room temperature to allow complex formation.

The siRNA-INTERFERin complexes were then added dropwise to the cells and gently mixed. Cells were incubated under standard culture conditions for 24 h, then 25 ng/ml PMA was added to differentiate cells to macrophages for another 24 h and they were rested for final extra 24 h. Knockdown efficiency was assessed by Western blot analysis 72 h after transfection. MISSION siRNA Universal Negative Control (SIC001-SIGMA) was used as a negative control to assess off-target effects and baseline expression.

Plasmid delivery (eYFP-TRPC3, eYFP-N-ter-TRPC3, eYFP-S712A- TRPC3, mCherry-PKCs and vYC4er cameleon) in HEK293-TLR4 cells was done using Lipofectamine 2000 (Thermo) or jetPRIME in THP-1 cells complexed with DNA in a 2:1 ratio respectively for 24–48 h.

*Trpc3* gene (gene ID:7223) in THP-1 cells was removed taking advantage of Synthego Express knockout cell pools high quality synthetic multiguide sgRNA technology (5′-UCCAGCAUCUUGCGCACCAC-3′; 5′-CGGCACCAGCCUCACGCCG-3′; and 5′-CUGUCAUGCGUCUCAGGGAU-3′) and transfected SpCas9. Editing efficiency was determined by T7 Endonuclease I (NEB) mismatch detection assay and confirmed by Sanger sequencing followed by ICE (Inference of CRISPR Edits) analysis (Synthego ICE tool). After limiting dilution selection of different clones, knockout of TRPC3 protein was validated by Western blot using anti-TRPC3 antibody (e.g., Alomone Labs, cat# ACC-016) and anti-β-actin as loading control. Macrophage function, including cytokine production, and phagocytic activity, was assessed post-editing to evaluate the impact of TRPC3 loss.

### 2.4 Lentiviral transduction

VSV-G–pseudotyped lentiviral particles were produced by co-transfection of 293FT cells with transfer constructs (eYFP-TRPC3 mutants) and the compatible packaging plasmids pMD2.G and psPAX2 in the presence of Lipofectamine 2000. Viruses were harvested at 48 and 72 hours after transfection. Lentiviral transduction of THP-1 macrophages was carried out using concentrated lentiviral particles in the presence of 8 μg/ml Polybrene (Sigma-Aldrich), and infected cells were selected by FACS sorting and subjected to qPCR.

### 2.5 Cytosolic Ca^2+^ measurement

Cells were loaded with 3 mM Fluo-4-AM for 20 min in culture medium at 37°C in a 5% CO_2_ incubator. Cells were then washed in indicator-free medium to remove any dye that was nonspecifically associated with the cell surface and then incubated for a further 20-min period to allow for complete hydrolysis of the acetoxymethyl esters. Live cell fluorescence was monitored by Leica Dmi8 system with an HC PL FLUOTAR 63X/1.3 NA oil immersion lens and an infrared focus maintenance system. For Fluo-4 imaging fluorescence was obtained using an excitation filter at 470/40 nm and an emission filter at 525/54 nm. Before imaging started, medium was replaced by HBSS supplemented with 10 mM HEPES, with 0 CaCl_2_ and 1.3 mM MgCl_2_. We recorded a 5 min baseline before adding LPS acquiring images each 5 s. and then restore extracellular calcium concentration by adding 1.3 mM CaCl_2_. Fluorescence data were analyzed using a combination routine of ImageJ and Cellpose semiautomated segmentation detection (15).

### 2.6 Live cell FRET imaging

Cells expressing FRET-based sensors (vYC4er or CKAR) were cultured on glass-bottom dishes and stimulated with LPS (1µg/mL). Imaging was performed at 37°C using a Leica Dmi8 system. Sensor localization was verified by YFP fluorescence. Ratiometric FRET/CFP and YFP signals were acquired with standard filter sets, and ratio images were generated using ImageJ as previously described (8, 14, 16).

### 2.7 FRET acceptor photobleaching

FRET efficiency between TRPC3 and PKC isoforms was assessed by acceptor photobleaching using a Leica confocal system as previously described (14). Briefly, mCherry was selectively photobleached in defined ROIs, and changes in eYFP fluorescence were quantified with ImageJ (AccPbFRET plugin). FRET efficiency was calculated using standard formulas, and appropriate positive and negative controls were included to confirm specificity (17).

### 2.8 qPCR

Total RNA was extracted using TRIzol reagent (Ambion). cDNA was obtained using Verso cDNA kit Reverse Transcription for RT-PCR (Thermo Fisher Scientific), following the manufacturer’s instructions. Quantitative PCR analysis was performed with an ABI7500 machine (Applied Biosystems) as previously described (18).

### 2.9 Immunoblots

Cells were lysed in Triton X-100 buffer containing protease and phosphatase inhibitors. Proteins were separated by SDS–PAGE, transferred to nitrocellulose membranes, and probed with specific primary antibodies followed by IRDye-conjugated secondary antibodies. Detection and quantification were performed using a LiCor Odyssey system as previously described (19, 20).

### 2.10 Quantification and statistical analysis

Statistical details of experiments are indicated in the figure legends. All data analyses were performed with Prism software (GraphPad). All data are presented as means ± standard error of the mean (SEM), indicating individual biological replicates. No statistical analysis was used to predetermine sample size. All datasets were analyzed by unpaired two-tailed Student’s t test. Welch’s correction was performed when the variances were significantly different.

## 3 Results

### 3.1 PKCα translocates to the plasma membrane while PKCϵ translocates to endomembranes after LPS stimulation

**Figure 1:**
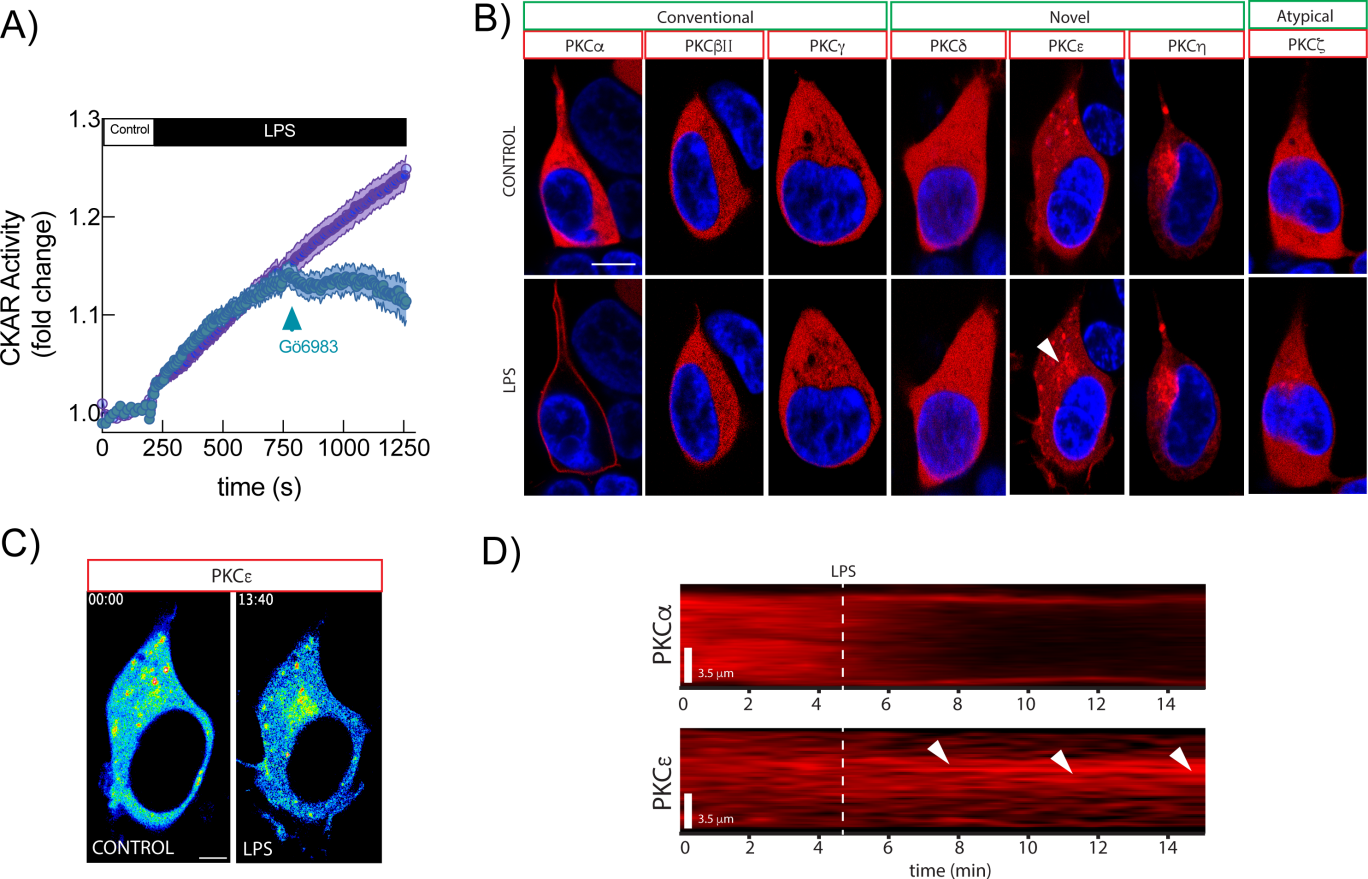
Subcellular localization of PKC isoforms in response to LPS. **(A)** HEK-TLR4 expressing CKAR were stimulated with 1 μg/ml LPS and Gö6983 was added to the media (blue trace) or not (purple trace) at the indicated time point. Fluorescence was analyzed by fluorescence microscopy. Mean FRET/CFP ratio is represented. **(B)** Live cell confocal microscopy localization analysis of the different PKC isoforms after 15 min exposure to LPS in HEK293-TLR4 cells expressing the various PKC isoforms fused to mCherry (red), with the nucleus stained in blue (Hoechst 33342). Representative images of n=5 are shown. **(C)** Pseudocolored analysis of PKCϵ translocation in response to LPS. **(D)** 2D Kymogram of a line crossing representative cells expressing PKCα or PKCϵ. Scale bar, 10 μm unless different indicated.

It has been proposed that pharmacological stimulation with PMA induces PKC-dependent phosphorylation of TRPC3 at S712 (7), leading to channel inactivation, we aimed to investigate whether specific PKC isoforms are responsible for this modification under more physiological conditions. As a first step, we assessed whether LPS stimulation induces PKC activity in HEK-TLR4 cells. To this end, we expressed the FRET-based PKC activity reporter CKAR (16), which is a pan PKC activity reporter composed by a monomeric FHA2 domain (forkhead-associated) from the yeast protein rad53p as a phospho-serine binding domain and a substrate consensus sequence predicted to be an excellent substrate for all PKC isoforms but suboptimal for other kinases (21, 22). Treatment of CKAR expressing HEK-TLR4 cells with LPS resulted in a ~35% rapid increase in PKC activity within 5 min, which was reduced to baseline by treatment with Gö6983 (**Figure 1A**), confirming PKC involvement in the response (23).

We next investigated the subcellular localization dynamics of PKC isoforms during LPS stimulation using live-cell confocal imaging of mCherry-tagged 7 out of 9 PKC isoforms. As shown in **Figures 1B–D**, only two isoforms exhibited stimulus-dependent translocation to membranous compartments. Specifically, PKCα translocated to the plasma membrane, whereas PKCϵ localized to intracellular endomembranes following LPS treatment (**Figure 1B**). The remaining PKC isoforms did not display detectable changes in subcellular distribution under these conditions.

### 3.2 PKCϵ interacts with TRPC3 in endomembranes

**Figure 2:**
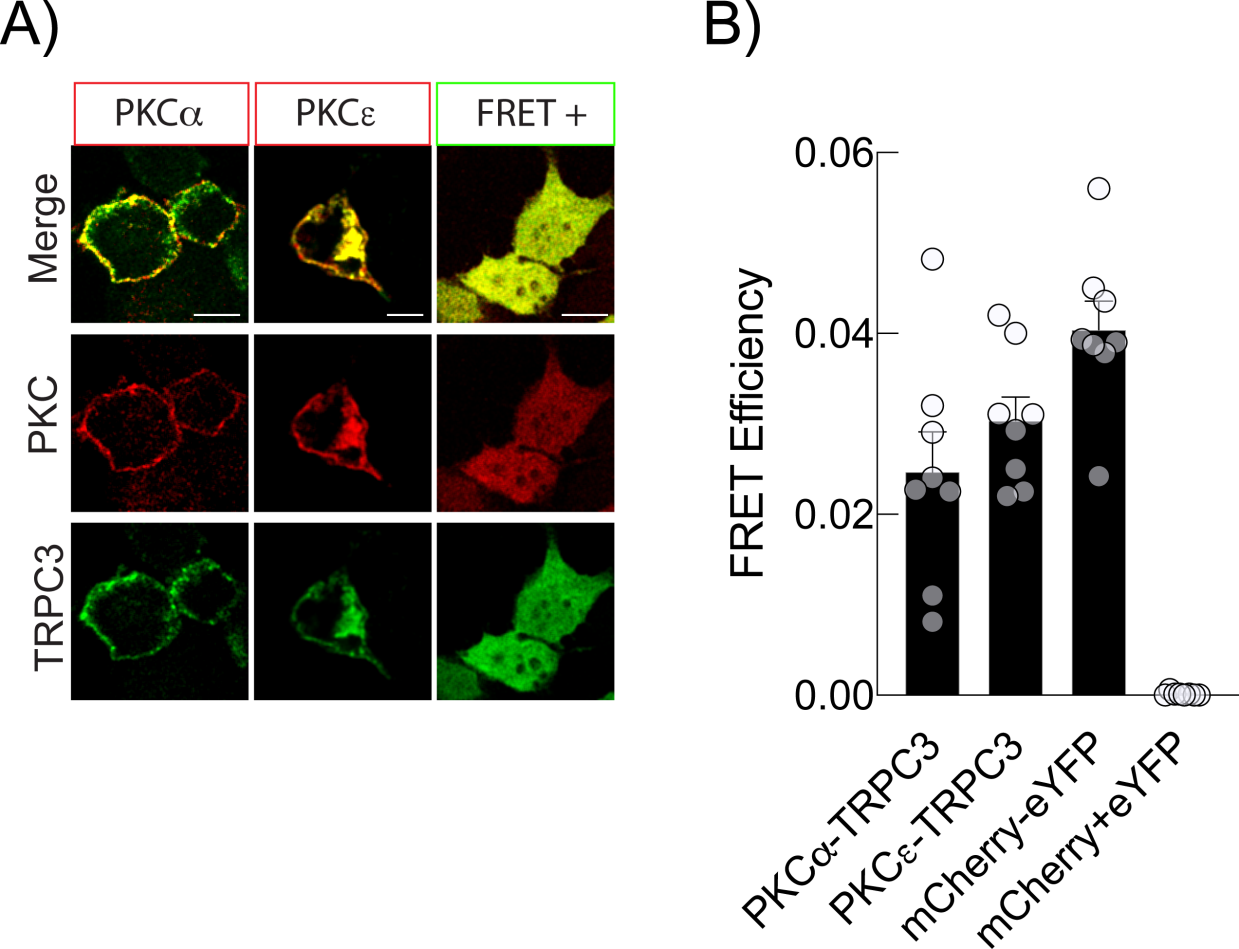
Colocalization and FRET analysis between PKCs and TRPC3. **(A)** Colocalization analysis of TRPC3-eYFP (green) and the PKCα-mCherry and PKCϵ-mCherry isoforms (red) using confocal microscopy and FRET assay in HEK-TLR4 cells stimulated with PMA for 10 min. **(B)** Bar graph showing FRET efficiency. Data are presented as means ± standard error from independent samples (n=8). Scale bar, 10 μm.

PKC activation is tightly regulated and typically short-lived, leading to rapid but transient phosphorylation of its substrates. This phosphorylation is often quickly reversed by cellular phosphatases, making it difficult to capture kinase/substrate interactions using traditional immunoprecipitation assays. In the case of TRPC3 and PKC, this transient nature—combined with the importance of subcellular localization—suggests that immunoprecipitation may not be the most suitable method for studying their interaction. As shown in **Figure 1**, subcellular redistribution of PKC isoforms appears to be a key regulatory mechanism during LPS stimulation. Then, we employed FRET- assays to monitor the interaction between TRPC3 and specific PKC isoforms in live cells. We co-expressed TRPC3-eYFP with either PKCα-mCherry or PKCϵ-mCherry, treated cells with PMA for 10 minutes to induce maximum membrane translocation of PKCs, and then analyzed FRET efficiency by acceptor photobleaching to assess proximity and potential interaction. The results showed that PKCα interacts with TRPC3 at the plasma membrane, whereas PKCϵ interacts with TRPC3 at endomembranes as compared with a FRET positive control consisting in an eYFP-mCherry fusion protein and a negative control of separately coexpressed eYFP and mCherry (**Figure 2**).

### 3.3 Inhibition of PKCϵ severely alter ER Ca^2+^ release in LPS stimulated cells

**Figure 3:**
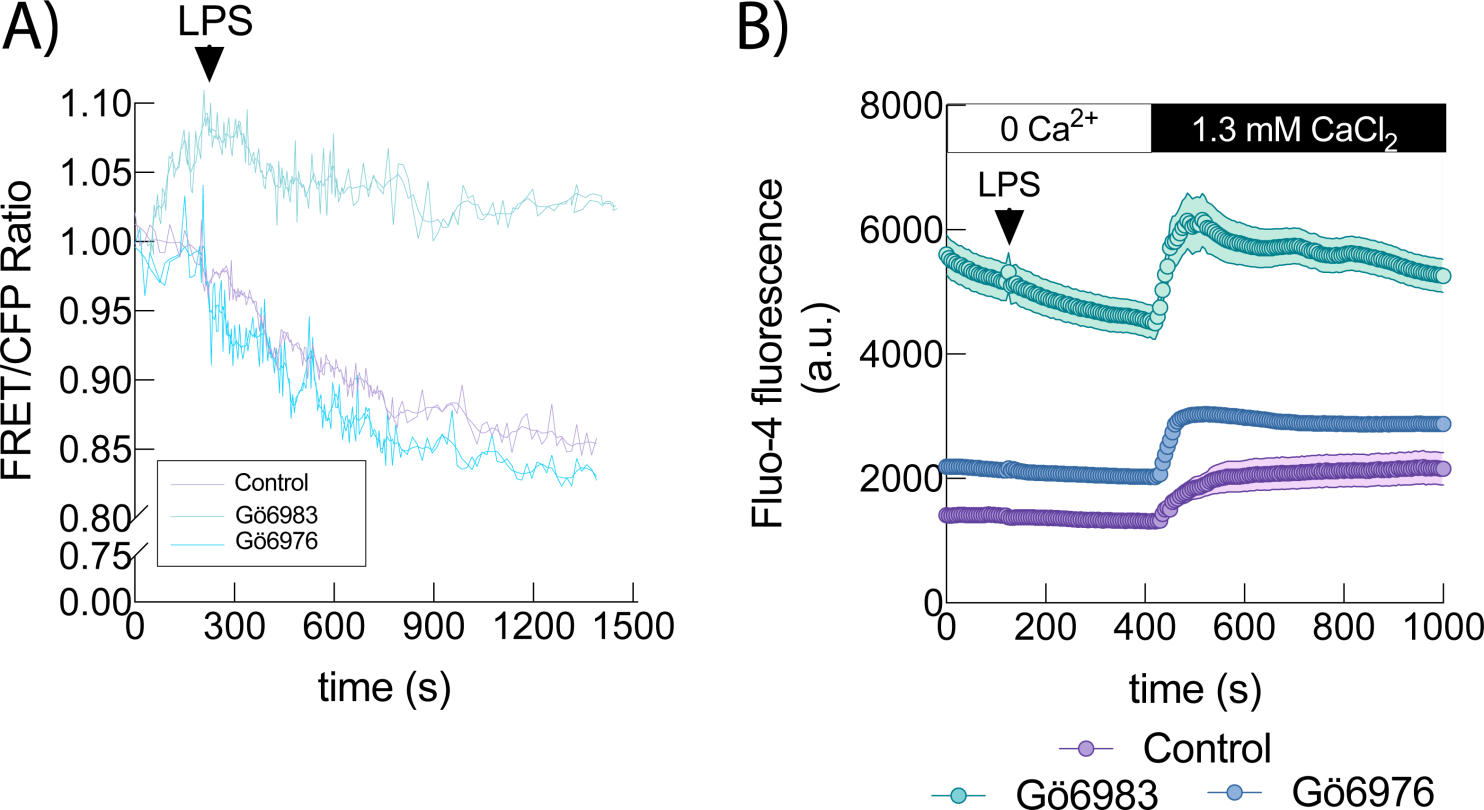
LPS-induced ER Ca^2+^ fluxes depend on novel PKCs activity. **(A)** HEK-TLR4 expressing the vCY4er calcium sensor, were treated with 500 nM Gö6976 (light blue; n =67 cells), 500 nM Gö6983 (light green; n = 52 cells) or vehicle (purple; n = 54 cells) and stimulated with 1 μg/ml LPS as indicated and fluorescence was analyzed by fluorescence microscopy. Mean FRET/CFP ratio is represented. **(B)** HEK-TLR4 cells pretreated with 500 nM Gö6976 (light blue; n =98 cells), 500 nM Gö6983 (light green; n = 97 cells) or vehicle (purple; n = 101 cells) were labeled with Fluo-4, and fluorescence was recorded before and after treating the cells with 1 μg/ml LPS, as indicated. Absolute changes in [Ca^2+^]i are shown (middle panel) or normalized (right panel).

As previous results confirmed a role in signaling for PKCα and ϵ during LPS stimulation, we next investigated ER Ca²^+^ dynamics under conditions of PKC inhibition. To distinguish between the roles of conventional and novel PKC isoforms, we utilized the selective inhibitors Gö6976, which targets conventional PKCs, and Gö6983, which inhibits both conventional and novel PKCs but not atypical isoforms (23–25). We employed a FRET-based calcium cameleon indicator (vYC4er), specifically engineered to localize within the endoplasmic reticulum (ER) lumen. This biosensor enables the detection of dynamic changes in ER [Ca²^+^] during cellular stimulation (26). HEK-TLR4 cells expressing the ER-targeted calcium sensor vYC4er were pretreated with either inhibitor or vehicle prior to LPS stimulation. Inhibition of conventional PKCs with Gö6976 had no significant effect on ER Ca²^+^ release following LPS stimulation. In contrast, treatment with Gö6983 caused ~35% higher basal ER Ca²^+^ and ~45% reduction in LPS-induced ER Ca²^+^ release compared to vehicle, similar to S712A cells (**Figure 3A**). Under extracellular Ca²^+^-free conditions, analysis of cytosolic Ca²^+^ dynamics revealed that cells pretreated with the pan-PKC inhibitor Gö6983 exhibited higher initial cytosolic Ca²^+^ levels compared to those treated with the classical PKC inhibitor Gö6976 or vehicle control. This elevated Ca²^+^ levels gradually declined during stimulation with LPS, suggesting active sequestration into endoplasmic reticulum (ER) stores (**Figure 3B**). These findings suggest that a novel PKC isoform, rather than a conventional one, mediates TRPC3 inactivation via phosphorylation. Among the novel PKCs examined by live-cell imaging, only PKCϵ exhibited stimulus-dependent translocation upon LPS treatment (**Figure 1**).

**Figure 4:**
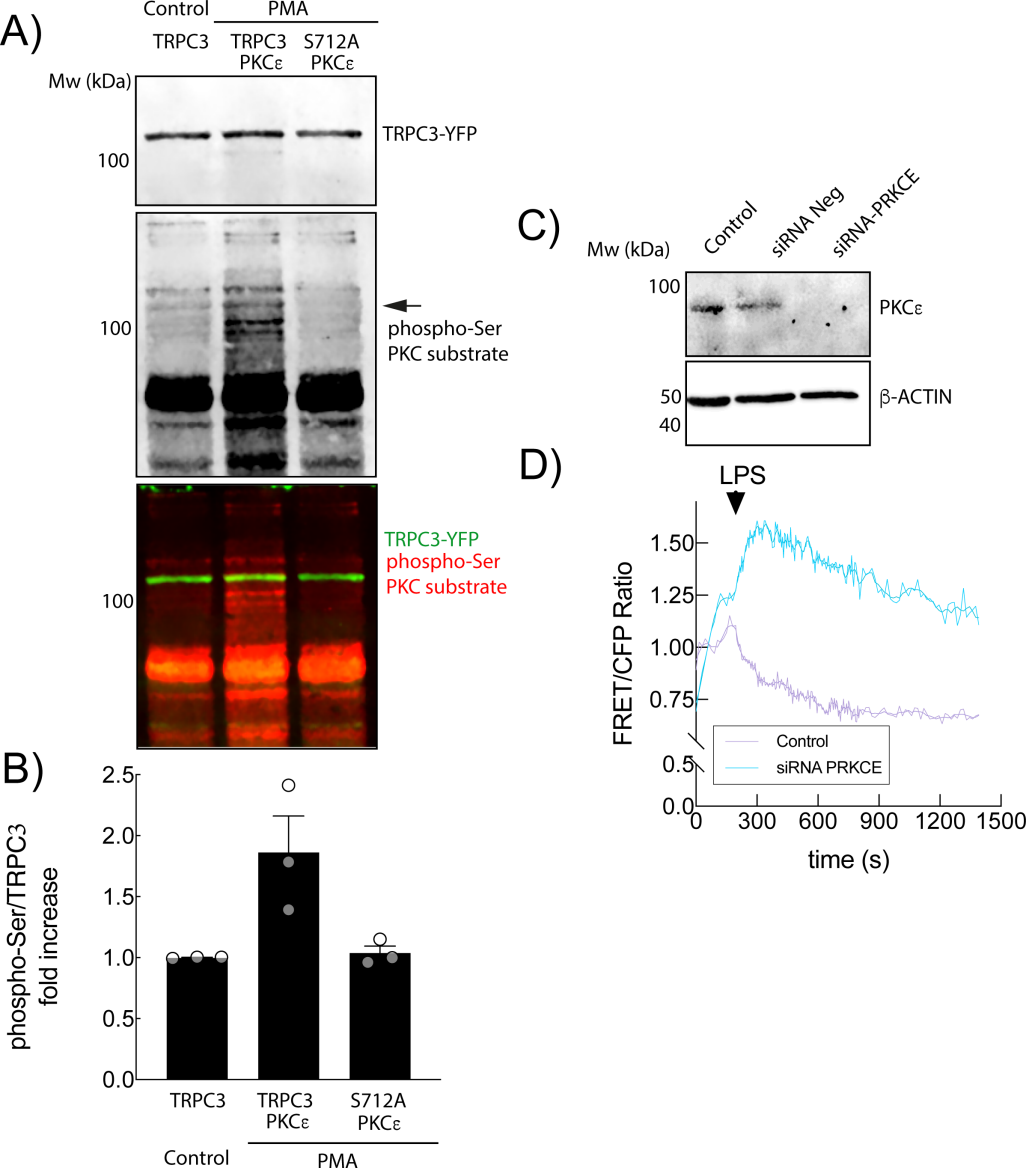
LPS-induced ER Ca^2+^ fluxes depend on PKCϵ activity. **(A)** HEK-TLR4 cells expressing TRPC3-WT, TRPC3-WT and PKCϵ or TRPC3-S712A mutant and PKCϵ were stimulated with PMA for 10 min to induce maximum PKC activation and analyzed by NIR Western blot with specific antibodies against TRPC3 (green) and phospho-Ser PKC substrates (red) **(B)** Fluorescence fold quantification of phospho-Ser PKC substrate (red) at the TRPC3-YFP molecular weight (green fluorescence). **(C)** THP-1 macrophages transfected with siRNA against *PRKCE* (light blue; n = 20 cells) or siRNA negative control (purple; n = 34 cells) were analyzed for PKCϵ expression by Western-blot using specific antibodies and β-ACTIN as a loading control. **(D)** Cells treated as in C and expressing the vCY4er calcium sensor, were stimulated with 200 ng/ml LPS as indicated and fluorescence was analyzed by fluorescence microscopy. The mean FRET/CFP ratio is represented.

To further explore the contribution of PKCϵ, we performed immunoblot analysis in HEK-TLR4 cells co-transfected with TRPC3-WT and PKCϵ. Using an anti-phospho-Ser substrate PKC antibody, we detected an increased phosphorylation signal in a protein band whose molecular weight is coincident with that of TRPC3 (**Figures 4A, B**). However, when TRPC3-S712A mutant and PKCϵ were co-expressed this phosphorylation increase was extremely reduced indicating that PKCϵ would be the isoform responsible for TRPC3-S712 phosphorylation. Based on this observation, we selectively silenced PKCϵ in human macrophages and monitored ER Ca²^+^ dynamics (**Figures 4C, D**). Consistent with pharmacological inhibition, PKCϵ knockdown resulted in a constitutive Ca²^+^ influx into the ER since the signal in the absence of PKCϵ is consistently much higher than in the control, indicating a greater Ca²^+^ content in the ER. Together, these results strongly implicate PKCϵ as the key isoform responsible for TRPC3 regulation via phosphorylation at serine 712 in response to LPS stimulation.

### 3.4 The phosphorylation of TRPC3 at Ser 712 alters its intracellular localization in HEK-TLR4 cells

**Figure 5:**
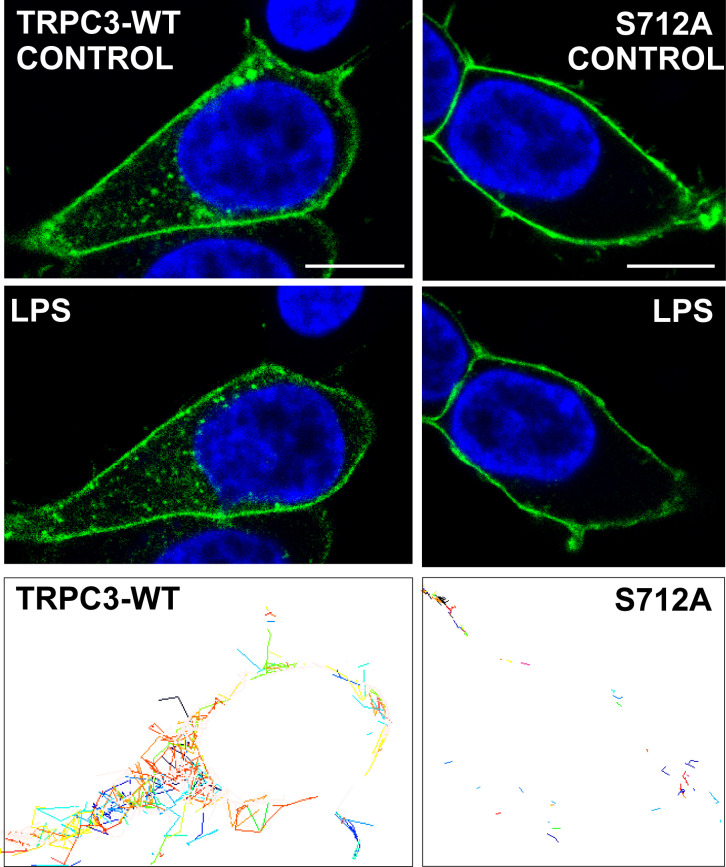
Subcellular distribution of TRPC3. HEK-TLR4 expressing either TRPC3-WT-eYFP or S712A-eYFP mutant were stimulated with 1μg/ml LPS and analyzed by live cell confocal microscopy. Nuclei were counterstained with Hoechst 33342. Bottom panes represent the tracks of the moving particles during LPS stimulation. Representative images of n=5 are shown. Scale bar, 10 μm.

Previous work suggested that TRPC3 is negatively regulated via phosphorylation at Ser 712 by PKC (7). To investigate the functional relevance of this post-translational modification, we expressed either eYFP-tagged TRPC3 wild-type (eYFP-TRPC3-WT) or a non-phosphorylatable mutant (eYFP-S712A-TRPC3), in which Ser 712 is substituted with Ala, in HEK-TLR4 cells (27). Live-cell confocal imaging revealed that, consistent with prior reports (8, 28) TRPC3-WT localized to both the plasma membrane and intracellular compartments. In contrast, the S712A mutant was exclusively localized to the plasma membrane (**Figure 5**). Following LPS stimulation, TRPC3-WT-expressing cells exhibited vesicular trafficking events, indicative of internalization or intracellular redistribution, which were absent in cells expressing the S712A mutant (**Figure 5**). These findings support the notion that phosphorylation at Ser 712 modulates TRPC3 subcellular localization and trafficking dynamics.

### 3.5 The phosphorylation of TRPC3 at Ser 712 affects the production of proinflammatory cytokines and the release of Ca^2+^ from the ER

**Figure 6:**
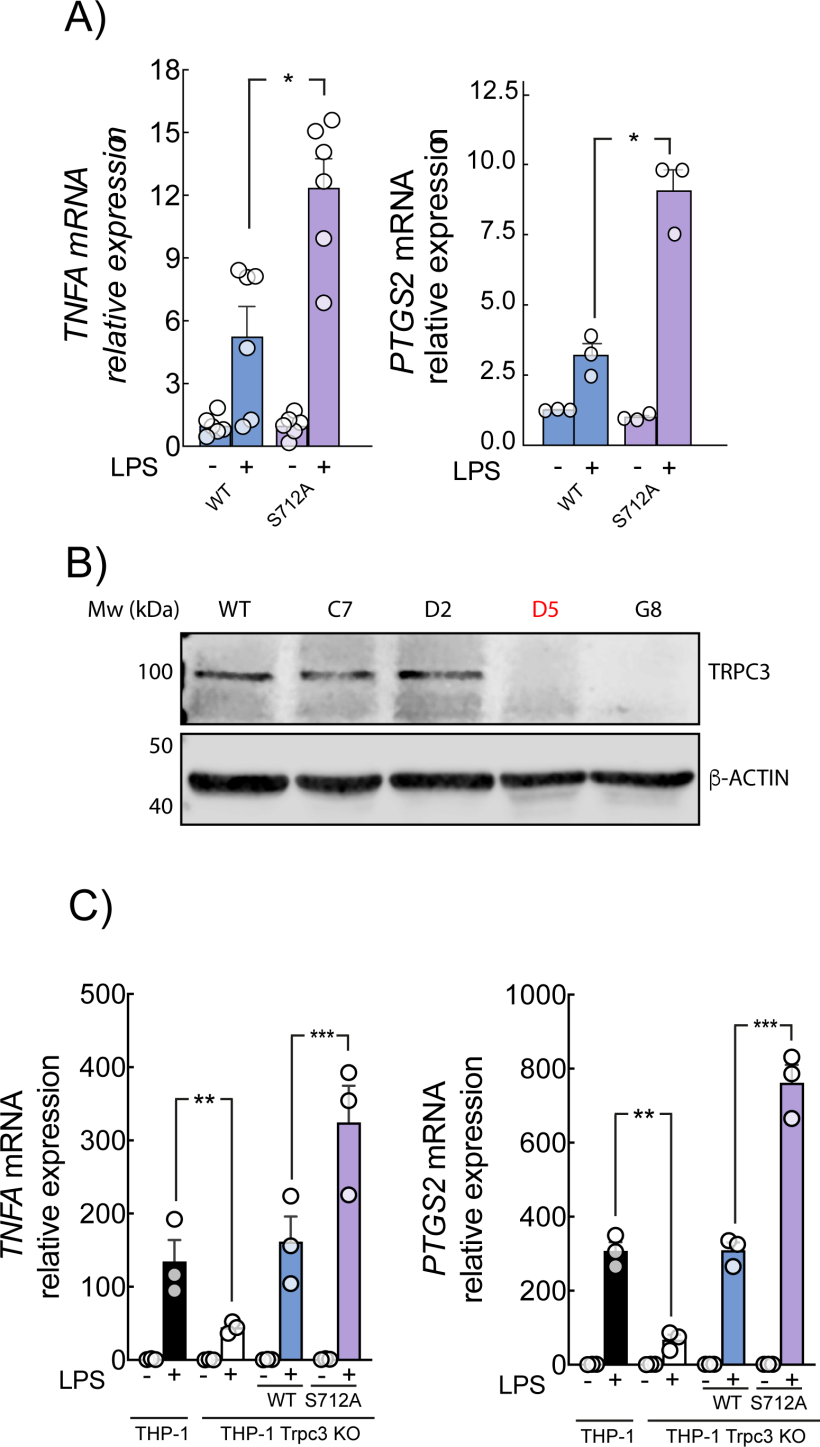
S712 phosphorylation is required to a correct TRPC3 signaling. **(A)** HEK-TLR4 cells ex-pressing TRPC3-WT or the mutant TRPC3- S712A were stimulated with 1 μg/ml LPS for 4 h and mRNA levels of the indicated genes analyzed by qPCR. **(B)** THP-1 WT cells and different *Trpc3* KO clones (C7, D2, D5 (selected for use) and G8) gene edited by CRISPR/Cas9-mediated gene editing followed by limiting dilution clonal selection were analyzed by NIR Western blot with specific antibodies against TRPC3. β-actin was used as loading control. **(C)** THP-1 cells (black) and THP-1 Trpc3 KO cells mock transduced (white), TRPC3-WT (blue) or TRPC3-S712A mutant (purple) were stimulated with 200 ng/ml LPS for 4 h and mRNA levels were analyzed as in **(A)** Error bars represent SEM (n=3). *p < 0.05; **p < 0.01; ***p < 0.001 by t-test.

To further explore the role of S712 phosphorylation of the TRPC3 channel, HEK-TLR4 cells were transfected with eYFP-TRPC3 or eYFP-S712A-TRPC3 and the production of proinflammatory cytokines was measured using qPCR. We observed that when TRPC3 could not be phosphorylated at S712, signaling effects are stronger, resulting in ~2.5-fold higher *TNF* and ~2-fold higher *PTGS2* expression compared to TRPC3-WT stimulated with LPS (**Figure 6A**). To confirm this effect in an immune relevant context, we took advantage of CRISPR/Cas9 technology and specifically deleted *TRPC3* gene in THP-1 macrophages (**Figure 6B**). In THP-1 macrophages, S712A reconstitution increased *TNF* by ~2.8-fold and *PTGS2* by ~2.2-fold relative to WT (**Figure 6C**).

**Figure 7:**
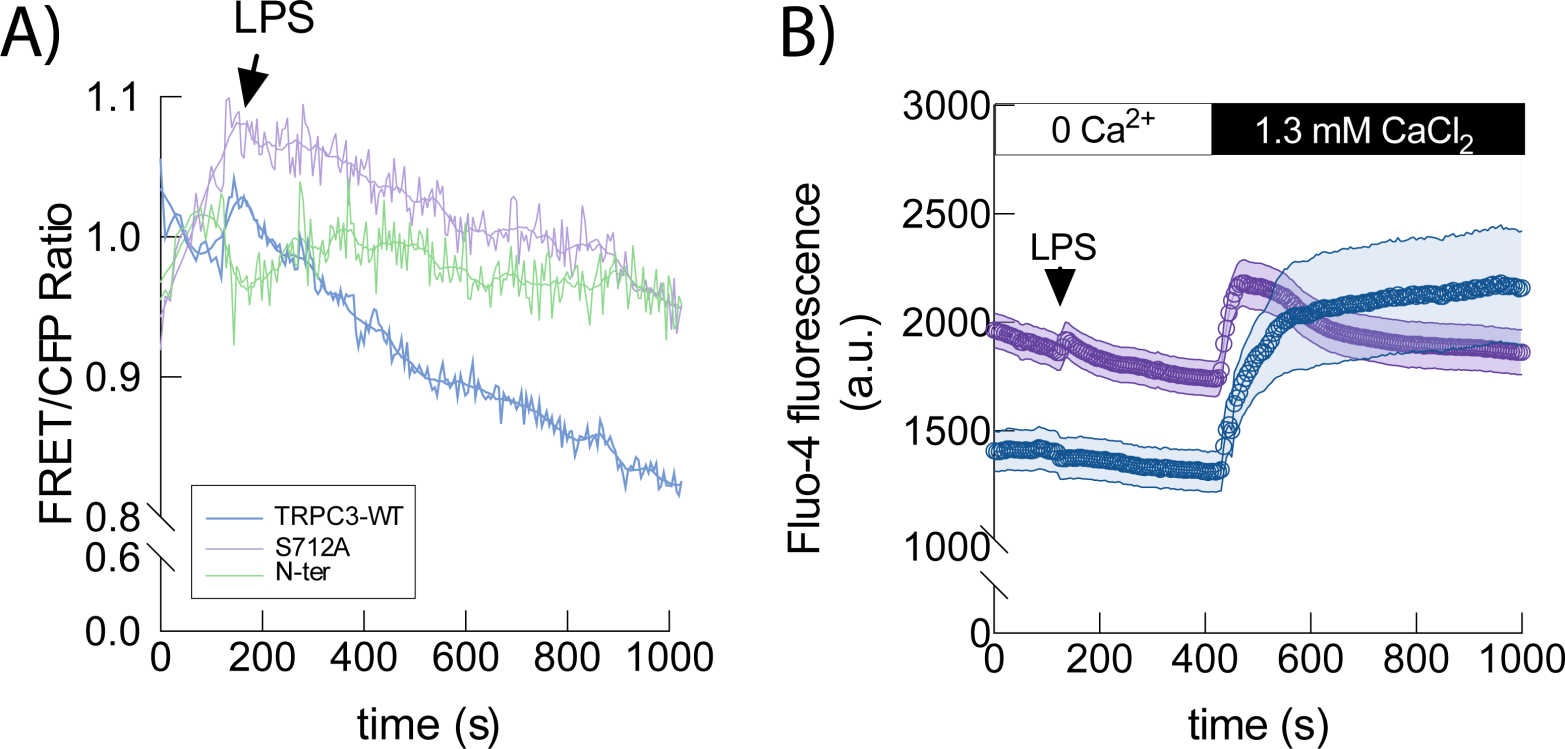
LPS-induced Ca^2+^ fluxes depend on S712 phosphorylation. **(A)** HEK-TLR4 expressing either TRPC3-WT (blue; n = 24 cells), the mutant S712A (purple; n = 26 cells) or the dominant negative N-ter fragment of TRPC3 (green; n= 25 cells) were transfected with the vCY4er calcium sensor, stimulated with 1 μg/ml LPS as indicated and fluorescence was analyzed by fluorescence microscopy. Mean FRET/CFP ratio is represented. **(B)** HEK-TLR4 cells expressing TRPC3-WT (blue) or the mutant S712A-TRPC3 (purple) were labeled with Fluo-4, and fluorescence was recorded before and after treating the cells with 1 μg/ml LPS, as indicated. Absolute changes in Fluo-4 fluorescence are shown (WT; n = 98 cells; S712A; n = 95 cells).

Previous research work from our lab has demonstrated the direct involvement of TRPC3 in the production of proinflammatory cytokines by LPS, a process that requires Ca^2+^ release from the ER (8). Therefore, we analyzed the dynamics of Ca^2+^ in the ER when TRPC3 could not be phosphorylated at S712. To that end, we co-expressed the ER targeted calcium cameleon vYC4er together with either TRPC3-WT or S712A-TRPC3. We observed that cells expressing TRPC3-WT exhibited Ca^2+^ release from the ER in response to LPS. This did not occur when cells expressed only the N-terminal fragment (302 aa), which acts as a dominant-negative preventing proper channel assembly. However, S712A-expressing cells showed ~30% higher basal ER Ca²^+^ levels and ~40% smaller LPS-induced ER Ca²^+^ release compared to WT (**Figure 7A**).

Consistent with previous findings, under extracellular Ca²^+^-free conditions, cells expressing the S712A mutant exhibited ~25% initial cytosolic Ca²^+^ compared to TRPC3-WT, which progressively declined over time, suggesting active sequestration into ER stores (**Figure 7B**) similar to the behavior observed in the Gö6983 treated cells in **Figure 3B**. Collectively, these findings suggest that phosphorylation of TRPC3 at S712 is necessary to contribute to the correct localization of the channel and to ensure the adequate Ca^2+^-mediated signaling responsible to produce proinflammatory cytokines.

## 4 Discussion

Our findings identify PKCϵ-mediated phosphorylation of TRPC3 at S712 as a key mechanism that terminates TRPC3-dependent Ca²^+^ signaling after TLR4 triggering in macrophages (29, 30). Across multiple complementary readouts—subcellular localization and trafficking, ER and cytosolic Ca²^+^ dynamics, cytokine production, PKC activity reporters, isoform translocation, FRET-based proximity assays, pharmacology/knockdown—we converge on a model in which LPS activates a spatially restricted PKCϵ pool on endomembranes that engages TRPC3 and inactivates the channel via S712 phosphorylation, thereby resolving Ca²^+^ signaling and tempering proinflammatory gene expression. This extends our previous work showing TRPC3 activation by diacylglycerol (DAG) and its role in macrophage inflammatory signaling (4, 8), and it provides isoform-level specificity to the earlier proposal that PKC phosphorylates TRPC3 at S712 to negatively regulate the channel in other cellular contexts (13).

Live-cell imaging revealed that TRPC3-WT subsets between the plasma membrane and intracellular membranes, with stimulus-evoked vesicular trafficking upon cellular activation with LPS, whereas the non-phosphorylatable S712A mutant is confined to the plasma membrane and fails to undergo LPS-induced redistribution. These observations suggest that phosphorylation at S712 is required for normal trafficking/endosomal residency of TRPC3 under inflammatory conditions. This interpretation aligns with a broader literature in which regulated exocytosis/endocytosis of TRP channels modulates their activity and cellular localization (31–34), and it provides a mechanistic refinement to earlier work that implicated S712 in their negative regulation without specifying the PKC isoform implicated on it or spatial context (13). Notably, overexpression of TRPC3 channels in HEK293 cells results in constitutively active channels in the plasma membrane perturbing Ca^2+^ homeostasis (28), an effect we also considered when interpreting our HEK–TLR4 data. The trafficking phenotypes we observe are therefore best understood in concert with our functional ER and cytosolic Ca²^+^ measurements and our THP1 reconstitution assays, which mitigate overexpression artifacts. Preventing TRPC3-S712 phosphorylation (TRPC3-S712A) led to sustained signaling and higher *TNF* and *PTGS2* induction in HEK–TLR4-TRPC3-WT expressing cells. The same trend held in THP1 macrophages lacking endogenous TRPC3 and reconstituted with TRPC3-WT or S712A. Functionally, TRPC3-WT supported LPS-evoked ER Ca²^+^ release, whereas TRPC3-S712A cells displayed constitutive Ca²^+^ entry into the ER and LPS responses that failed to appreciably alter ER Ca²^+^ from its elevated baseline. In parallel, TRPC3-S712A cells exhibited elevated basal cytosolic Ca²^+^ under Ca²^+^-free conditions that decayed as ER stores refilled, and they showed altered Ca²^+^ influx upon Ca²^+^ re-addition compared with WT during LPS treatment. Taken together, these results indicate that S712 phosphorylation is necessary to its correct localization and the TRPC3-dependent Ca²^+^ signaling homeostasis after LPS in line with previous work from our group linking lipin1–derived DAG to intracellular TRPC3 activation and inflammatory signaling (8). Based on the available evidence, both transient and sustained Ca^2+^ increases can lead to TNFα production, depending on the stimulus and pathway involved. It has been demonstrated that LPS causes a transient Ca^2+^ increase and concluded that the transient increase of Ca^2+^ plays a role in LPS-induced expression of TNFα (29, 35). However, a sustained Ca^2+^ elevation mediated through a phospholipase-A_2_-dependent pathway, might be essential for induction of TNFα secretion in response to acetylated LDL (30, 36).

Using the CKAR pan-PKC activity reporter, we confirmed that LPS increases PKC activity in HEK–TLR4 cells and that this activity is stopped by the broad PKC inhibitor Gö6983. These observations are concordant with reports that TLR signaling engages PKC pathways in macrophages (37–39). However, live-cell imaging uncovered a decisive spatial distinction: following LPS, PKCα translocated to the plasma membrane, whereas PKCϵ translocated to endomembranes. This subcellular segregation—rather than intrinsic catalytic selectivity—likely dictates which isoform functionally engages TRPC3 during inflammatory signaling in living cells. Of note, since PKC-ϵ can be phosphorylated by PKCα, it is possible that the effects of PKCα on cytokine secretion are shared by PKCϵ (39, 40).

FRET experiments established proximity between TRPC3 and PKCϵ on endomembranes, whereas TRPC3–PKCα proximity was confined to the plasma membrane. Complementarily, pharmacological inhibition that includes novel PKCs (Gö6983) mimics the S712A mutant behavior in ER and cytosolic Ca²^+^ assays, while inhibition of conventional PKCs (Gö6976) did not. Selective knockdown of PKCϵ in THP1 macrophages reproduced the Gö6983 and TRPC3-S712A phenotypes. These data support PKCϵ as the physiologically relevant isoform phosphorylating TRPC3 at S712 and with our earlier work placing TRPC3 activation within a lipin1–DAG–rich intracellular environment (8).

Based on these findings, we propose the following model: Upon LPS–TLR4 engagement, lipin-1–derived DAG activates intracellular TRPC3, promoting ER Ca²^+^ release and cytosolic Ca²^+^ elevation that supports inflammatory gene induction in macrophages (4–8, 30, 37). Concurrently, PKCϵ relocates to endomembranes, encounters TRPC3, and phosphorylates S712. This phosphorylation inactivates TRPC3, restores Ca²^+^ homeostasis, and restrains cytokine production. Thus, PKCϵ-mediated S712 phosphorylation functions as a critical off-switch for TRPC3-driven Ca²^+^ signaling during macrophage activation (16, 38, 39). Importantly, the TRPC3 phosphorylation detected in macrophages in this study should be considered part of the intrinsic negative regulatory mechanisms that limit inflammation.

## Data Availability

The raw data supporting the conclusions of this article will be made available by the authors, without undue reservation.

## References

[1] Rayees S Joshi JC Tauseef M Anwar M Baweja S Rochford I . PAR2-mediated cAMP generation suppresses TRPV4-dependent ca2+ Signaling in alveolar macrophages to resolve TLR4-induced inflammation. Cell Rep. (2019) 27:793–805.e4. doi: 10.1016/j.celrep.2019.03.053, PMID: 30995477 PMC6485424

[2] Arfath Y Kotra T Faizan MI Akhtar A Abdullah ST Ahmad T . TRPV4 facilitates the reprogramming of inflamed macrophages by regulating IL-10 production via CREB. Inflammation Res. (2024) 73:1687–97. doi: 10.1007/s00011-024-01923-3, PMID: 39101955

[3] Kumarasamy S Solanki S Atolagbe OT Joe B Birnbaumer L Vazquez G . Deep transcriptomic profiling of M1 macrophages lacking trpc3. Sci Rep. (2017) 7:39867. doi: 10.1038/srep39867, PMID: 28051144 PMC5209678

[4] Hofmann T Obukhov AG Schaefer M Harteneck C Gudermann T Schultz G . Direct activation of human TRPC6 and TRPC3 channels by diacylglycerol. Nature. (1999) 397:259–63. doi: 10.1038/16711, PMID: 9930701

[5] Balboa MA de Pablo N Meana C Balsinde J . The role of lipins in innate immunity and inflammation. Biochim Biophys Acta - Mol Cell Biol Lipids. (2019) 1864:1328–37. doi: 10.1016/j.bbalip.2019.06.003, PMID: 31220616

[6] Zhang P Reue K . Lipin proteins and glycerolipid metabolism: Roles at the ER membrane and beyond. Biochim Biophys Acta. (2017) 1859:1583–95. doi: 10.1016/j.bbamem.2017.04.007, PMID: 28411173 PMC5688847

[7] Lutkewitte AJ Finck BN . Regulation of signaling and metabolism by lipin-mediated phosphatidic acid phosphohydrolase activity. Biomolecules. (2020) 10:1386. doi: 10.3390/biom10101386, PMID: 33003344 PMC7600782

[8] Casas J Meana C López-López JR Balsinde J Balboa MA . Lipin-1-derived diacylglycerol activates intracellular TRPC3 which is critical for inflammatory signaling. Cell Mol Life Sci. (2021) 78:8243–60. doi: 10.1007/s00018-021-03999-0, PMID: 34757442 PMC8629864

[9] Meana C García-Rostán G Peña L Lordén G Cubero Á Orduña A . The phosphatidic acid phosphatase lipin-1 facilitates inflammation-driven colon carcinogenesis. JCI Insight. (2018) 3:e97506. doi: 10.1172/jci.insight.97506, PMID: 30232275 PMC6237220

[10] Meana C Pena L Esquinas E Lorden G Guijas C Valdearcos M . Lipin-1 integrates lipid synthesis with proinflammatory responses during TLR activation in macrophages. J Immunol (Baltimore Md: 1950). (2014) 193:4614–22. doi: 10.4049/jimmunol.1400238, PMID: 25252959

[11] Valdearcos M Esquinas E Meana C Gil-de-Gómez L Guijas C Balsinde J . Subcellular localization and role of lipin-1 in human macrophages. J Immunol. (2011) 186:6004–13. doi: 10.4049/jimmunol.1003279, PMID: 21478406

[12] Newton AC . Protein kinase C: perfectly balanced. Crit Rev Biochem Mol Biol. (2018) 53:208–30. doi: 10.1080/10409238.2018.1442408, PMID: 29513138 PMC5901981

[13] Trebak M . Negative regulation of TRPC3 channels by protein kinase C-mediated phosphorylation of serine 712. Mol Pharmacol. (2004) 67:558–63. doi: 10.1124/mol.104.007252, PMID: 15533987

[14] Hochreiter B Kunze M Moser B Schmid JA . Advanced FRET normalization allows quantitative analysis of protein interactions including stoichiometries and relative affinities in living cells. Sci Rep-uk. (2019) 9:8233. doi: 10.1038/s41598-019-44650-0, PMID: 31160659 PMC6547726

[15] Stringer C Wang T Michaelos M Pachitariu M . Cellpose: a generalist algorithm for cellular segmentation. Nat Methods. (2021) 18:100–6. doi: 10.1038/s41592-020-01018-x, PMID: 33318659

[16] Violin JD Zhang J Tsien RY Newton AC . A genetically encoded fluorescent reporter reveals oscillatory phosphorylation by protein kinase C. J Cell Biol. (2003) 161:899–909. doi: 10.1083/jcb.200302125, PMID: 12782683 PMC2172956

[17] Roszik JSJ Vereb G . AccPbFRET: An ImageJ plugin for semi-automatic, fully corrected analysis of acceptor photobleaching FRET images. BMC Bioinf. (2008) 9:346. doi: 10.1186/1471-2105-9-346, PMID: 18713453 PMC2571114

[18] Valdearcos M Esquinas E Meana C Peña L Gil-de-Gómez L Balsinde J . Lipin-2 reduces proinflammatory signaling induced by saturated fatty acids in macrophages. J Biol Chem. (2012) 287:10894–904. doi: 10.1074/jbc.m112.342915, PMID: 22334674 PMC3322858

[19] Casas J Meana C Esquinas E Valdearcos M Pindado J Balsinde J . Requirement of JNK-mediated phosphorylation for translocation of group IVA phospholipase A2 to phagosomes in human macrophages. J Immunol. (2009) 183:2767–74. doi: 10.4049/jimmunol.0901530, PMID: 19625654

[20] Casas J Brzostek J Zarnitsyna VI Hong J Wei Q Hoerter JAH . Ligand-engaged TCR is triggered by Lck not associated with CD8 coreceptor. Nat Commun. (2014) 5:5624. doi: 10.1038/ncomms6624, PMID: 25427562 PMC4248239

[21] Nishikawa K Toker A Johannes F-J Songyang Z Cantley LC . Determination of the specific substrate sequence motifs of protein kinase C isozymes*. J Biol Chem. (1997) 272:952–60. doi: 10.1074/jbc.272.2.952, PMID: 8995387

[22] Yaffe MB Leparc GG Lai J Obata T Volinia S Cantley LC . A motif-based profile scanning approach for genome-wide prediction of signaling pathways. Nat Biotechnol. (2001) 19:348–53. doi: 10.1038/86737, PMID: 11283593

[23] Gschwendt M Dieterich S Rennecke J Kittstein W Mueller H-J Johannes F-J . Inhibition of protein kinase C μ by various inhibitors. Inhibition from protein kinase c isoenzymes. FEBS Lett. (1996) 392:77–80. doi: 10.1016/0014-5793(96)00785-5, PMID: 8772178

[24] Martiny-Baron G Kazanietz MG Mischak H Blumberg PM Kochs G Hug H . Selective inhibition of protein kinase C isozymes by the indolocarbazole Gö 6976. J Biol Chem. (1993) 268:9194–7. doi: 10.1016/s0021-9258(18)98335-3, PMID: 8486620

[25] Kim S-Y Kim S-W Kim J-M Jho E-H Park S-Y Oh D-Y . PKC inhibitors RO 31–8220 and Gö 6983 enhance epinephrine-induced platelet aggregation in catecholamine hypo-responsive platelets by enhancing Akt phosphorylation. BMB Rep. (2011) 44:140–5. doi: 10.5483/bmbrep.2011.44.2.140, PMID: 21345315

[26] Miyawaki A Llopis J Heim R McCaffery JM Adams JA Ikura M . Fluorescent indicators for Ca2+ based on green fluorescent proteins and calmodulin. Nature. (1997) 388:882–7. doi: 10.1038/42264, PMID: 9278050

[27] Medvedev AE Vogel SN . Overexpression of CD14, TLR4, and MD-2 in HEK 293T cells does not prevent induction of *in vitro* endotoxin tolerance. J endotoxin Res. (2003) 9:60–4. doi: 10.1179/096805103125001360, PMID: 12691621

[28] Löf C Blom T Törnquist K . Overexpression of TRPC3 reduces the content of intracellular calcium stores in HEK-293 cells. J Cell Physiol. (2008) 216:245–52. doi: 10.1002/jcp.21396, PMID: 18431718

[29] Watanabe N Suzuki J Kobayashi Y . Role of calcium in tumor necrosis factor-alpha production by activated macrophages. J Biochem. (1996) 120:1190–5. doi: 10.1093/oxfordjournals.jbchem.a021540, PMID: 9010769

[30] Qi H-Y Shelhamer JH . Toll-like receptor 4 signaling regulates cytosolic phospholipase A2 activation and lipid generation in lipopolysaccharide-stimulated macrophages*. J Biol Chem. (2005) 280:38969–75. doi: 10.1074/jbc.m509352200, PMID: 16176925

[31] Bezzerides VJ Ramsey IS Kotecha S Greka A Clapham DE . Rapid vesicular translocation and insertion of TRP channels. Nat Cell Biol. (2004) 6:709–20. doi: 10.1038/ncb1150, PMID: 15258588

[32] Cayouette S Lussier MP Mathieu E-L Bousquet SM Boulay G . Exocytotic insertion of TRPC6 channel into the plasma membrane upon Gq protein-coupled receptor activation. J Biol Chem. (2004) 279:7241–6. doi: 10.1074/jbc.m312042200, PMID: 14662757

[33] Morenilla-Palao C Planells-Cases R García-Sanz N Ferrer-Montiel A . Regulated exocytosis contributes to protein kinase C potentiation of vanilloid receptor activity. J Biol Chem. (2004) 279:25665–72. doi: 10.1074/jbc.m311515200, PMID: 15066994

[34] Singh BB Lockwich TP Bandyopadhyay BC Liu X Bollimuntha S Brazer S-C . VAMP2-dependent exocytosis regulates plasma membrane insertion of TRPC3 channels and contributes to agonist-stimulated Ca2+ influx. Mol Cell. (2004) 15:635–46. doi: 10.1016/j.molcel.2004.07.010, PMID: 15327778

[35] Zhou X Yang W Li J . Ca 2+ - and protein kinase C-dependent signaling pathway for nuclear factor-κB activation, inducible nitric-oxide synthase expression, and tumor necrosis factor-α Production in lipopolysaccharide-stimulated rat peritoneal macrophages. J Biol Chem. (2006) 281:31337–47. doi: 10.1074/jbc.m602739200, PMID: 16923814

[36] Pollaud-Chérion C Vandaele J Quartulli F Séguélas M Decerprit J Pipy B . Involvement of calcium and arachidonate metabolism in acetylated-low-density-lipoprotein-stimulated tumor-necrosis-factor-α production by rat peritoneal macrophages. Eur J Biochem. (2022) 253:345–53. doi: 10.1046/j.1432-1327.1998.2530345.x, PMID: 9578494

[37] Letari O Nicosia S Chiavaroli C Vacher P Schlegel W . Activation by bacterial lipopolysaccharide causes changes in the cytosolic free calcium concentration in single peritoneal macrophages. J Immunol (Baltimore Md: 1950). (1991) 147:980–3. doi: 10.4049/jimmunol.147.3.980, PMID: 1907309

[38] Loegering DJ Lennartz MR . Protein kinase C and toll-like receptor signaling. Enzyme Res. (2011) 2011:1–7. doi: 10.4061/2011/537821, PMID: 21876792 PMC3162977

[39] Shapira L Sylvia VL Halabi A Soskolne WA Dyke TEV Dean DD . Bacterial lipopolysaccharide induces early and late activation of protein kinase C in inflammatory macrophages by selective activation of PKC-ϵ. Biochem Biophys Res Commun. (1997) 240:629–34. doi: 10.1006/bbrc.1997.7717, PMID: 9398616

[40] Saurin AT Durgan J Cameron AJ Faisal A Marber MS Parker PJ . The regulated assembly of a PKCε complex controls the completion of cytokinesis. Nat Cell Biol. (2008) 10:891–901. doi: 10.1038/ncb1749, PMID: 18604201

